# Supplementary material for: Physiological responses of Oxyrrhis marina to a diet of virally infected Emiliania huxleyi
Source: PeerJ. 2019 Apr 19;7:e6722. doi: 10.7717/peerj.6722 (PMC6476294; doi:10.7717/peerj.6722)
Supplement: Supplemental Information 1 [file peerj-07-6722-s001.pdf]

Table S1. *Emiliania huxleyi* N and C content (pg/cell)

Treatment	Time (hours p.i.)	Replicate	pg N/cell	pg C/cell	Mean ± SD pg N/cell	Mean ± SD pg C/cell
Eh inf	0	A	1.580	9.190	1.507 ± 0.077	9.268 ± 0.1898
		B	1.550	9.580		
		C	1.500	9.036		
		D	1.555	9.165		
		E	1.366	9.368		
		F	1.488	9.270		
	24	A	1.945	11.102	1.889 ± 0.129	10.856 ± 0.430
		B	1.755	10.758		
		C	1.782	10.991		
		D	2.093	10.216		
		E	1.816	10.607		
		F	1.945	11.461		
Eh non-inf	0	A	1.480	8.559	1.507 ± 0.050	8.940 ± 0.936
		B	1.573	8.242		
		C	1.434	8.988		
		D	1.551	10.778		
		E	1.511	8.681		
		F	1.494	8.394		
	24	A	2.053	10.686	2.088 ± 0.072	10.415 ± 0.258
		B	1.997	10.410		
		C	2.110	10.714		
		D	2.212	10.173		
		E	2.088	10.427		
		F	2.067	10.082		

Emiliania huxleyi non-infected (Eh non-inf); *Emiliania huxleyi* infected with EhV-86 (Eh inf). Time indicate hours post-infection. Values are mean ± one standard deviation.

Table S2. *Oxyrrhis marina*'s average growth (day^{-1}) and grazing rates (Eh cells Om^{-1} h^{-1} (experiment1) or Eh cells Om^{-1} day^{-1} (experiments 2 – 4) for each experiment under both diet conditions.

	**Experiment 1**		**Experiment 2**		**Experiment 3**		**Experiment 4**	
	**Om + Eh non-inf**	**Om + Eh inf**	**Om + Eh non-inf**	**Om + Eh inf**	**Om + Eh non-inf**	**Om + Eh inf**	**Om + Eh non-inf**	**Om + Eh inf**
Growth	NA	NA	0.433 ± 0.020	0.563 ± 0.025	0.325 ± 0.005	0.466 ± 0.006	0.2826 ± 0.068	0.540 ± 0.052
Grazing	14.880 ± 1.570	14.480 ± 0	57.873 ± 1.067	41.163 ± 2.545	50.877 ± 2.106	25.241 ± 0.648	72.263 ± 6.963	52.821 ± 2.760
Growth/Grazing	NA	NA	0.0075 ± 0.0001	0.0140 ± 0.0008	0.0068 ± 0.0005	0.0231 ± 0.0004	0.0051 ± 0.0010	0.0130 ± 0.0008

Oxyrrhis marina (Om); *Emiliania huxleyi* non-infected (Eh non-inf); *Emiliania huxleyi* infected with EhV-86 (Eh inf). Values are mean ± one standard deviation.

Table S3. *Oxyrrhis marina*'s growth (day^{-1}) and grazing rates (Eh cells Om^{-1} day^{-1} or Bact cells Om^{-1} day^{-1}) during experiment 5.

	**Experiment 5**									
	**Om + Eh inf**					**Om + < 0.4 µm filt**				
**Day**	**1**	**2**	**3**	**4**	**Average**	**1**	**2**	**3**	**4**	**Average**
Growth	0.57 ± 0.02	0.34 ± 0.05	0.11 ± 0.06	0.49 ± 0.11	0.38 ± 0.02	0.04 ± 0.43	0.40 ± 0.42	0.06 ± 0.19	nd	0.10 ± 0.05
Grazing *E.huxleyi*	49.4 ± 1.56	60.8 ± 2.07	93.3 ± 5.02	56.2 ± 2.86		3.5 ± 4.04	nd	nd	nd	
Grazing Bacteria	226 ± 64.51	nd	nd	nd		767 ± 130.34	nd	3010 ± 869.17	3132 ± 455.02	

Oxyrrhis marina (Om); *Emiliania huxleyi* infected with EhV-86 (Eh inf); < 0.4 µm filtrate of *Emiliania huxleyi* culture infected with EhV-86 (Om + < 0.4 µm filt). Daily average values are mean of biological replicates for each day ± one standard deviation. "Average" values are the mean of the biological replicates ± one standard deviation of the averages of the biological replicates.

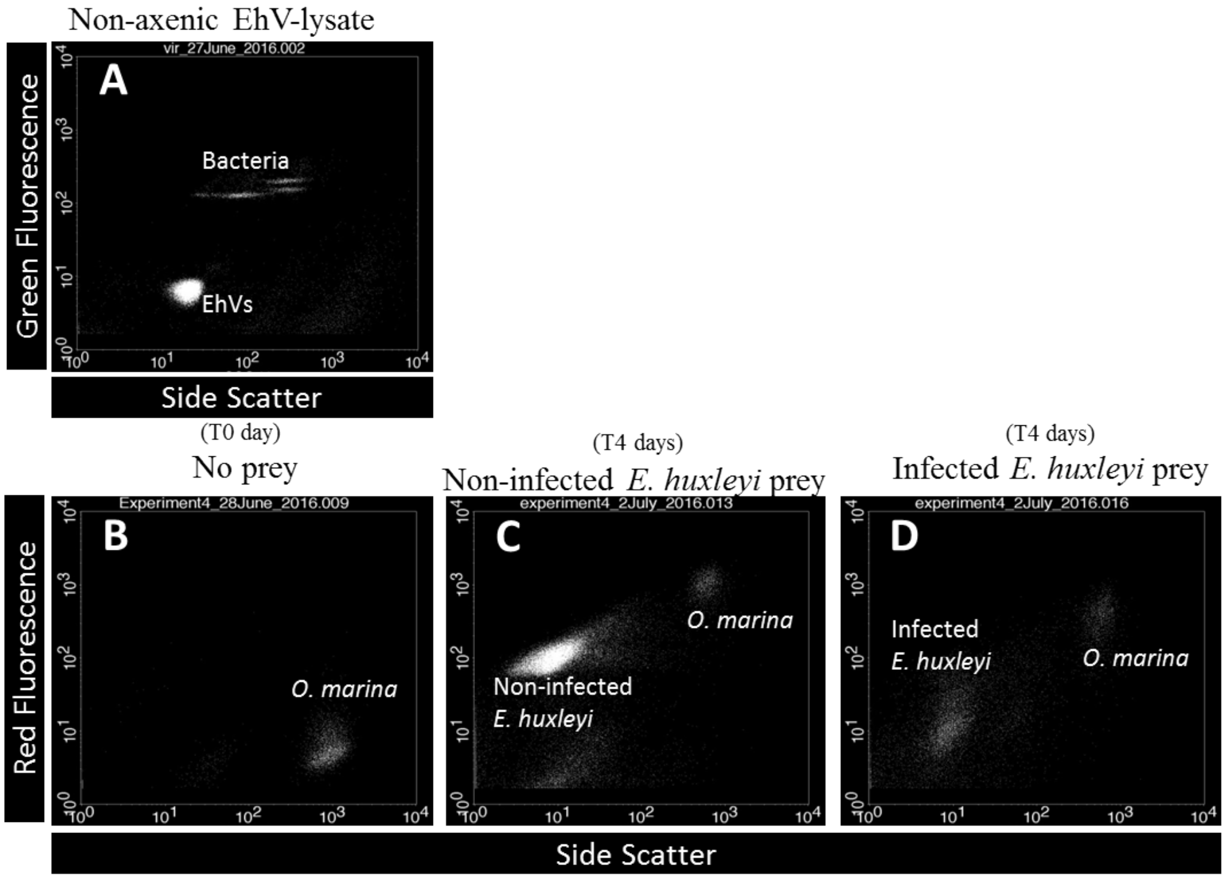

Fig. S1. Representative biparametric flow cytometry dotplots from Exp. 4, showing populations of **A:** *E. huxleyi* viruses (EhVs) and bacteria, based on SYBR Green I fluorescence *versus* side scatter; **B:** *O. marina* 3-day starved at the beginning of the experiment (T0), based on chlorophyll fluorescence *versus* side scatter; **C:** *O. marina* and non-infected *E. huxleyi*, based on chlorophyll fluorescence *versus* side scatter, on the last experimental day (T4); **D:** *O. marina* and virally infected *E. huxleyi*, based on chlorophyll fluorescence *versus* side scatter, on the last experimental day (T4).

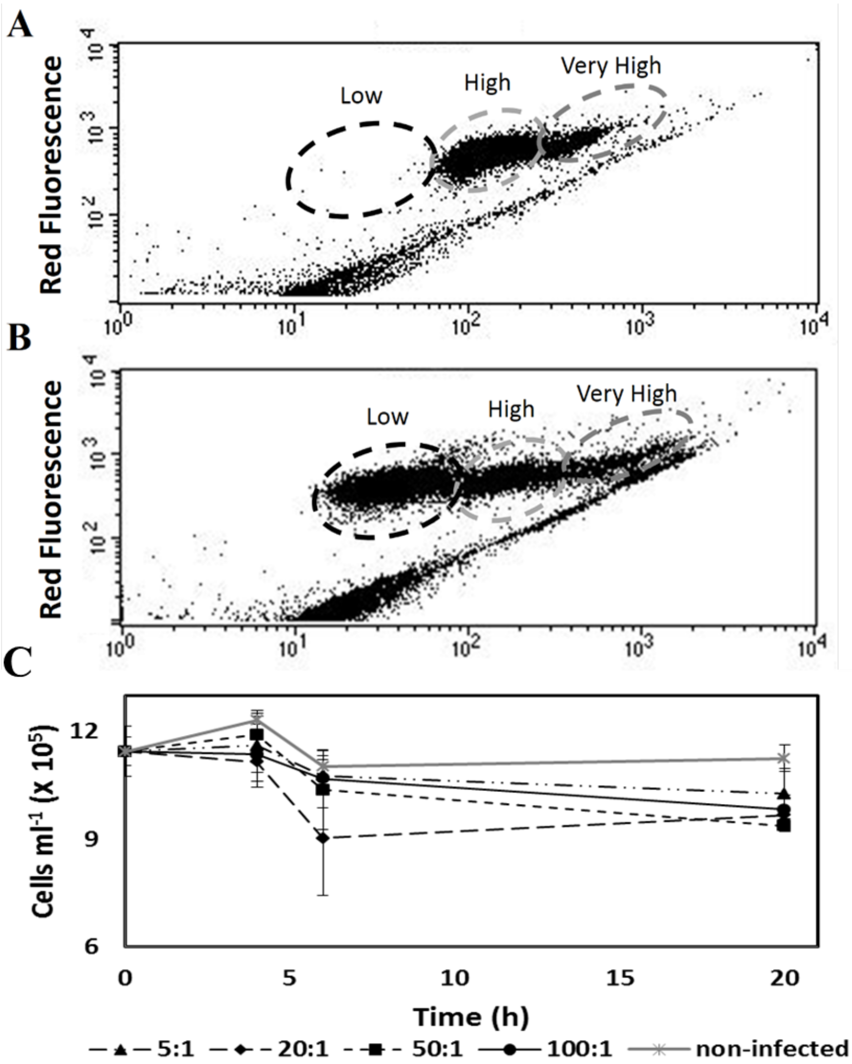

Fig. S2. **A:** Non-infected *E. huxleyi* and **B:** EhV infected *E. huxleyi*representative FCM dotplots of *E. huxleyi* stained with lipid fluorescent dye FM1-43 20 h p.i. X-axis is relative orange fluorescence and Y-axis is relative red fluorescence. Higher abundances of cells exhibiting a very high or low orange fluorescence compared to non-infected cells are considered visibly infected. **C:** Total *E. huxleyi* cell abundance over a 20 h period of time in non-infected cultures and in cultures virally infected at virus:host ratios of 5:1, 20:1, 50:1, and 100:1. Biological triplicates were enumerated for each treatment. Values are mean ± one standard deviation.

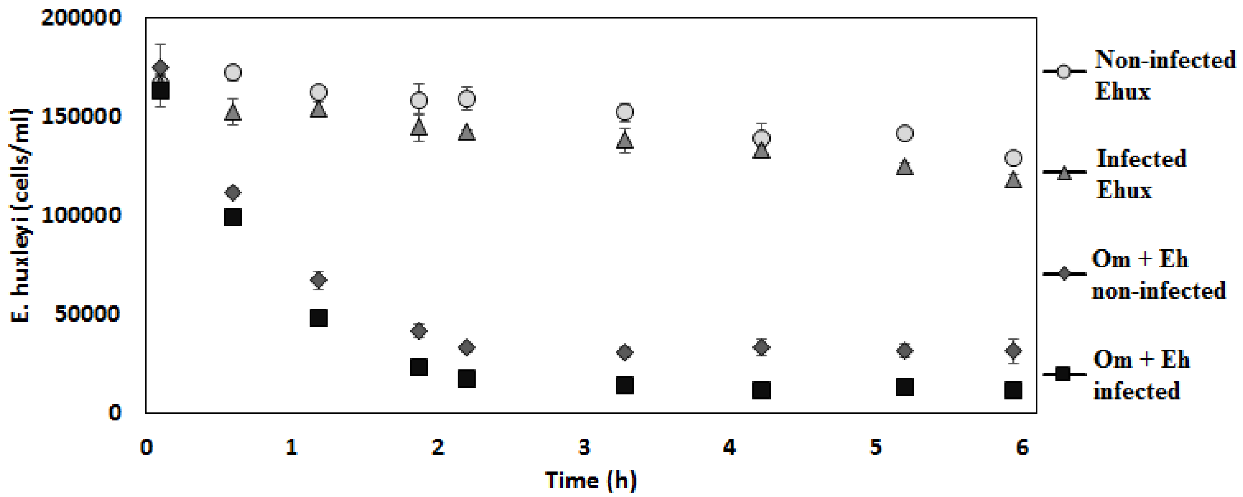

Fig. S3. *E. huxleyi* concentration (cells ml^{-1}) over time (hours) during experiment 1. *Oxyrrhis marina* (Om); *Emiliania huxleyi* (Eh). Values are mean ± one standard deviation. Measurable grazing occurred during the initial 1.9 h p.i.

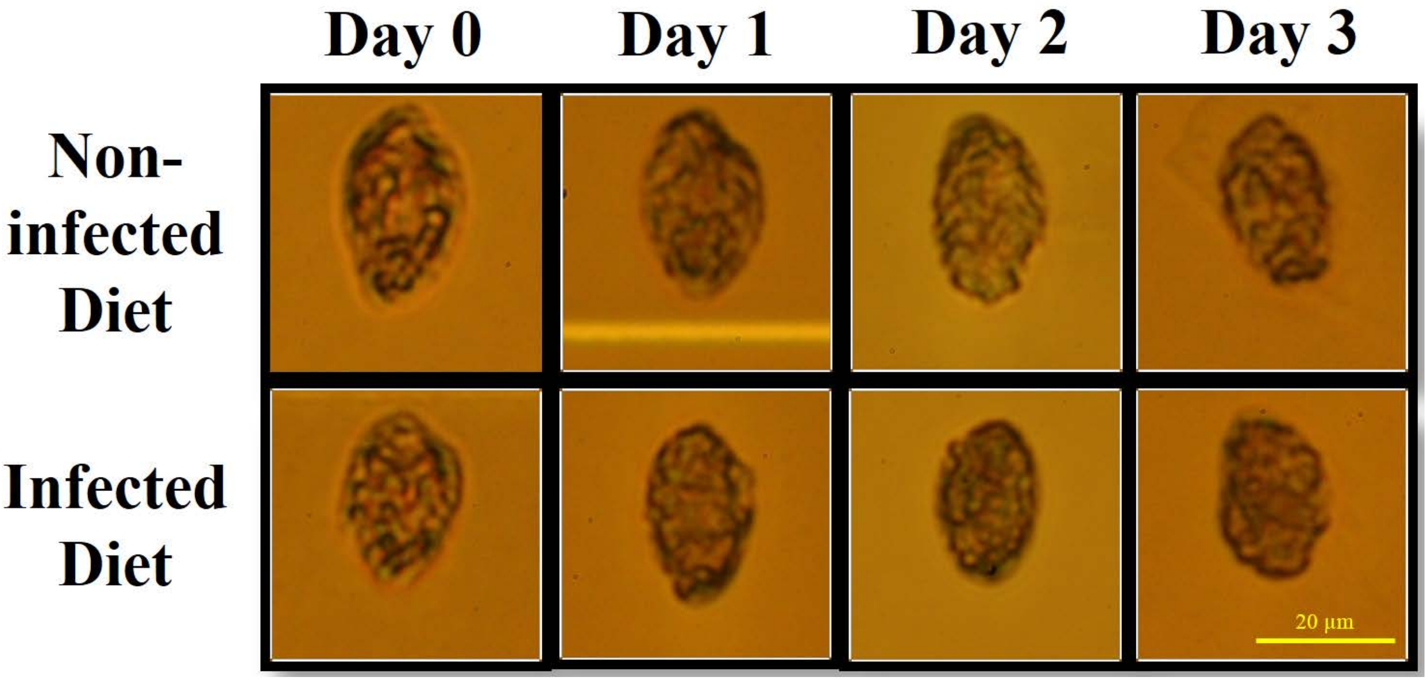

Fig. S4. Representative *O. marina* pictures taken for volume measurements.
